# Notes and News

**Published:** 1896-05-09

**Authors:** 


### NOTES AND NEWS.

(Collected and Communicated.)

### HOSPITAL MEETINGS.

A Festival Dinner in aid of the National Orthopædic Hospital will be held at the Whitehall Rooms, Hotel Metropole, on Thursday, May 14th, under the presidency of the Duke of Marlborough.

A Festival Dinner in aid of the North London Consumption Hospital will be held at the Hotel Cecil on June 9th, the Right Hon. Lord Kinnaird presiding.

The Annual Meeting of Governors will be held at the Metropolitan Hospital, Kingsland Road, N.E., on Monday, May 18th, at 2 p.m.

Count Mattei, whose name is known in connection with a professed remedy for cancer, died recently at Bologna.

It is intended to erect a new operating theatre at the Grimsby Hospital, to enlarge the servants' quarters, and reconstruct the drainage.

The Princess of Wales has fixed Thursday, May 21st, for the opening of the bazaar and *fête* to be held in the new wing and grounds of the West London Hospital.

The Society of French Physicians has decided to organise a fund for the relief of the widows and orphans of medical men in France.

The Merchant Taylors' Company has made a special grant of twenty guineas towards the rebuilding fund of the Dental Hospital of London.

The Prince of Wales, on the afternoon of the 5th inst., paid an unexpected visit to Guy's Hospital, and spent an hour and a-half in inspecting the wards, museums, and residential college, with the medical and dental schools.

June 6th is appointed for Hospital Saturday at Birmingham, and £14,000 is asked for. The people of Birmingham are justly proud of their fund, and the confidence with which the managers ask for the sum required and the willing manner in which the public respond might well provoke emulation elsewhere.

The New York Homœopathic Medical College has been presented by ex-Governor Flower with a sum of money to endow five beds for the treatment of patients, and Mrs. Kundhart has endowed a ward in connection with the college.

Mrs. Keeley, the veteran actress, has given £28 to the re-endowment fund of Guy's Hospital, the remaining sum of the proceeds of a matinée held in her honour in November, all of which she distributed in charity.

The Hospital for Epilepsy and Paralysis, Regent's Park, N.W., has been fortunate enough to secure from the executors of the late David James an allotment of £450 out of the legacy left by him for the benefit of Jewish and other charities.

Messrs. N. M. Rothschild and Sons have sent 50 guineas to the Royal Chest Hospital, City Road, towards the fund being raised in connection with the festival dinner to be held at the Hotel Metropole on May 26th.

The Russian Committee of the Twelfth International Medical Congress are fulfilling a general wish by placing the English language on the same basis as the German. French will be the official language of the congress.

The Duke of Newcastle has accepted the chairmanship of the committee connected with the bazaar to be opened by the Duchess of Connaught at the Queen's Hall, Langham Place, on June 23rd, in aid of the North-Eastern Hospital for Children, Hackney Road, Shoreditch.

At the recent meeting of the Royal Alfred Merchant Seamen's Institution, it was stated that for the next election of pensioners there were 250 applicants, the majority being over 70 years of age. Of this large number only 13 can be benefited, three being admitted into the home and the remainder receiving pensions. Some of the candidates have been up for as many as twenty elections.

This week's mail brings us an account of the opening by the Governor of Victoria of the new wing of the Victorian Eye and Ear Hospital. This wing completes the hospital buildings, and has cost upwards of £4,000. The proceedings included the presentation of an address to Mrs. Aubrey Bowen, through whose munificence in supplementing the legacy left by her husband, the late Dr. Aubrey Bowen, the new wing has been erected and furnished.

We hope the managers of the Nottingham General Hospital will see their way to carry out the suggestion made by Mr. F. Ley at the annual meeting held on the 22nd ult., that the accounts for the in and out patient departments should be given separately. That this is most necessary all who have had to prepare statistics relating to the work of our hospitals have had great cause to know. The Nottingham General Hospital, if it only adopts this suggestion, will be setting an excellent example to other institutions all over the country.

